# Association between CSF1 and CSF1R Polymorphisms and Parkinson’s Disease in Taiwan

**DOI:** 10.3390/jcm8101529

**Published:** 2019-09-24

**Authors:** Kuo-Hsuan Chang, Yih-Ru Wu, Yi-Chun Chen, Hsiu-Chuan Wu, Chiung-Mei Chen

**Affiliations:** Department of Neurology, Chang Gung Memorial Hospital-Linkou Medical Center, Chang Gung University College of Medicine, Taipei 333, Taiwan; gophy5128@cgmh.org.tw (K.-H.C.); yihruwu@cgmh.org.tw (Y.-R.W.); asd108@cgmh.org.tw (Y.-C.C.); serenawu@cgmh.org.tw (H.-C.W.)

**Keywords:** *CSF1*, *CSF1R*, neuroinflammation, Parkinson’s disease, polymorphism, disease association

## Abstract

Background: CSF1/CSF1R neuroinflammatory signaling is emerging as an important pathway involved in the pathogenesis of Parkinson’s disease (PD). However, the genetic associations between *CSF1*/*CSF1R* and PD have not yet been explored. Methods: We investigated the effects of two functional genetic variants, including *CSF1* rs1058885 and *CSF1R* rs10079250 in a cohort including 502 Taiwanese patients with PD and 511 age- and gender-matched healthy controls. Results: The *CSF1* rs1058885 TT genotype was less frequent in PD patients compared with control subjects (odds ratio (OR) = 0.63, 95% confidence interval (CI): 0.43–0.92, *p* = 0.015). The PD patients also had a lower frequency of the *CSF1* rs1058885 T allele compared with the control subjects (OR = 0.80, 95% CI: 0.67–0.96, *p* = 0.014). No statistically significant differences in allelic and genotypic frequencies of *CSF1R* rs10079250 between the PD and control subjects were found, even after stratification by age at onset and gender. Conclusion: This study reports a genetic association between *CSF1* and PD for the first time.

## 1. Introduction

Parkinson’s disease (PD), characterized by rigidity, resting tremor, and slow movement, is a common neurodegenerative disease [1]. The pathogenesis of PD is mainly associated with the degeneration of dopaminergic (DAergic) neurons and the presence of inclusion bodies enriched with α-synuclein in the substantia nigra (SN) of the ventral midbrain [2]. Neuroinflammation, which has been repeatedly observed in PD pathology, may contribute to PD pathogenesis and is thought to be a therapeutic target for PD [3]. Neuroinflammation has been shown to interact with α-synuclein aggregation via a vicious cycle, in which microglia are activated, which produce pro-inflammatory cytokines, chemokines, and complements, leading to the overt production of reactive oxygen species (ROS) and nitrogen species, and eventually resulting in neuronal loss [4].

Microglia, the most important part of the defense system against pathogens in the brain and spinal cord, constitute around 10% of cells in the central nervous system [5]. Proliferation, survival, and polarization of microglia are mediated by CSF1 and its receptor, CSF1R [6]. CSF1R-deficient mice have defective microglial development and demonstrate severe microglial loss [7,8]. Inhibition of CSF1R depletes microglia in the central nervous system [9]. In contrast, overexpression of *CSF1* promotes microglial proliferation in transgenic mouse models [10]. In addition to proliferation regulation of microglia, the CSF1/CSF1R-mediated pathway also plays a role in the functional phenotype of microglia. When CSF1 was injected into mouse brains, the inflammatory response of microglia against lipopolysaccharides (LPS) was impaired [11]. These findings indicate the pivotal role of CSF1/CSF1R signaling in neuroinflammation.

It has been suggested that genetic variants of *CSF1* and *CSF1R* are associated with different inflammatory diseases, such as asthma and periodontitis [12,13,14]. However, the roles of genetic variants of *CSF1* and *CSF1R* in PD have not yet been revealed. In this study, we assessed the potential association of two functional genetic variants, namely *CSF1* rs1058885 [15] and *CSFR1* rs10079250 [16], of PD via a case-control association study in 502 PD patients and 511 age- and gender-matched healthy control subjects in Taiwan.

## 2. Subjects and Methods

### 2.1. Ethics Statement

This study was approved by the institutional review boards of Chang Gung Memorial Hospital (ethical license No: 102-5614A3 and 201701921A3). Written informed consent was obtained from all participants.

### 2.2. Patient Population

We recruited 1013 subjects, including 502 patients (female/male: 253/249) with PD and 511 normal control subjects (female/male: 252/259) in the neurology clinics of Chang Gung Memorial Hospital-Linkou Medical center (Table 1). The mean age at onset of PD symptoms was 63.64 ± 10.76 years (range 19–89), and that of control subjects upon recruitment was 63.38 ± 11.90 years (range 26–94). The diagnosis of PD was made according to the UK Brain Bank diagnostic criteria by 3 movement disorder-specialized neurologists (YR Wu, CM Chen, and KH Chang) [17]. The disease stage was evaluated using the Hoehn and Yahr scale [18].

Among the disease group, 13 patients presented with a family history of PD, while 489 patients were sporadic. To avoid the skew caused by multiple family members carrying the same genetic variants, we only included one proband if patients had a family history of PD. None of the patients had Sjőgren syndrome, systemic lupus erythromatosus, rheumatoid arthritis, vasculitis, or malignancy. Unrelated healthy volunteers with matched ages, genders, ethnic origins, and areas of residence were enrolled as the control subjects. Patients with an age of onset of ≤50 years were categorized as early-onset PD (EOPD), while those with an age of onset of >50 years were categorized as late-onset PD (LOPD).

### 2.3. Genetic Analysis

Functional single nucleotide polymorphisms (SNPs) in *CSF1* and *CSF1R*, such as rs1058885 and rs10079250 [15,16], may influence the signaling pathways and the inflammatory process response involving PD neurodegeneration. Therefore, we genotyped these two SNPs to assess their potential associations with PD. The *CSF1* rs1058885 polymorphism was examined using the Agena MassARRAY platform with iPLEX gold chemistry SNP (San Diego, CA, USA). Genomic DNA of peripheral leukocytes was extracted using a DNA Extraction Kit (Stratagene, La Jolla, CA, USA). The specific PCR primer for rs1058885 genotyping (forward: ACGTTGGATGTGTGGCTGAGCAGAGAGGGT, reverse: ACGTTGGATGCCAGGCTCTCCCAGGATCT) and extension primers (CCCCACCCAGGATCTCATCAC) were designed using the Assay Designer software package (v.4.0, Agena, San Diego, CA, USA). Briefly, 10 ng DNA was loaded with 5 μL of the PCR reaction mixture, containing 500 nmol of each PCR primer mix, 1 unit of Taq polymerase, and 2.5 mM of each deoxy-ribonucleoside triphosphate (Agena, San Diego, CA, USA). The thermocycling reaction was set at 94 °C for 4 min, followed by 45 cycles of 94 °C for 20 sec, 56 °C for 30 sec, and 72 °C for 1 min, then 72 °C for 3 min. Shrimp alkaline phosphatase (0.3 U) was added to deactivate unincorporated dNTP. A single base extension reaction was performed using iPLEX enzyme, terminator mix, and extension primer mix, under thermocycling of 94 °C for 30 sec followed by 40 cycles of 94 °C for 5 sec, and 5 cycles of 56 °C for 5 sec and 80 °C for 5 sec, then 72 °C for 3 min (iPLEX gold kit, Agena, San Diego, CA, USA). Subsequently, 7 nL of the primer extension reaction, which was purified by cation exchange resin, was loaded onto the matrix pad of a SpectroCHIP (Agena, San Diego, CA, USA). MassARRAY Analyzer 4 (Agena, San Diego, CA, USA) was used to analyze the SpectroCHIPs.

The *CSF1R* rs10079250 polymorphism was genotyped using a custom-designed TaqMan SNP genotyping assay (assay ID: C__22274425_20) using the ABI 7000 Real Time PCR system (Applied Biosystems, Foster City, CA, USA). Briefly, 20 ng of DNA was loaded with 5 μL of the PCR reaction mixture with 0.9 µM of each primer, 0.2 µM of probe (probe sequence: GCGGGGCAGAGAGAGGGTGAAGGTG[C/T]GCCTGCAGGAGAGAATCAGGTGGTG), and Universal PCR Master Mix (Applied Biosystems). Thermocycling was set at 50 °C for 2 min, 95 °C for 10 min, 40 cycles of 95 °C for 15 sec, and finally 60 °C for 1 min. The results were analyzed using SDS software version 1.1 (Applied Biosystems, Foster City, CA, USA).

### 2.4. Statistical Analysis

The distribution of both genotypes did not deviate from the Hardy–Weinberg equilibrium. The allele and genotypic frequencies between the PD patients and the controls were compared using the Chi square test. The level of statistical significance was set at *p* < 0.025 (two tailed) to adjust multiple comparisons. Given the observed allele frequency at the significance level of 0.025, we had a power of greater than 0.8 when the odds ratio (OR) of the per-allele genetic effect was greater than 1.4 or lesser than 0.70 for both the rs1058885 and rs10079250 variants.

## 3. Results

The *CSF1* rs1058885 TT genotype was less frequent in PD patients compared with the control subjects (OR = 0.63, 95% confidence interval (CI): 0.43–0.92, *p* = 0.015, Table 2). PD patients had a lower frequency of the *CSF1* rs1058885 T allele compared with the control subjects (OR = 0.80, 95% CI: 0.67–0.96, *p* = 0.014). Further stratification according to the age at symptom onset and gender did not find significant differences in the allelic and genotypic frequencies of *CSF1* rs1058885 between PD patients and the control subjects.

The allelic and genotypic frequencies of *CSF1R* rs10079250 were similar in both the PD and control groups (Table 3). There were no statistically significant differences in the allelic and genotypic frequencies between PD patients and control subjects after stratification of age of symptom onset and gender.

## 4. Discussion

This study showed that the *CSF1* rs1058885 polymorphism affects the risk of PD in the Taiwanese population. This is the first study to identify a specific genotype of *CSF1* associated with PD. The results strongly support the role of neuroinflammation in PD neurodegeneration, with *CSF1* rs1058885 playing a particular role in this process.

*CSF1* rs1058885 is associated with aggressive periodontitis in the Japanese population [14]. The T allele in rs1058885 may reduce the risk of periodontitis. However, this association cannot be replicated in Han-Chinese patients with chronic periodontitis [12]. The genetic association between rs1058885 and Alzheimer’s disease was studied in the European population, but the result was negative [19]. It is important to note that rs1058885 is located within a highly variable region of exon 6 of *CSF1*. The frequency of the minor rs1058885 T allele in our study (44.1%) was similar to East Asian population (40%), whereas this allele is predominant in American (67%), African (54%), European (66%), and South Asian (65%) populations according to 1000 Genome (http://www.1000genomes.org/home). To determine whether *CSF1* rs1058885 is associated with PD in different ethnic populations, more genetic studies should be performed in different races.

CSF1 acts as a mitogen of microglia [20,21]. Microglial proliferation can be induced by systemic delivery or direct injection of CSF1 into the hippocampus of mouse models used to investigate prion disease [22], Alzheimer’s disease [23], ischemic stroke [24], and amyotrophic lateral sclerosis [24]. In *CSF1*-overexpressing mice, inhibition of CSF1R reversed the increase in the number of microglia by promoting microglial apoptosis [10]. On the other hand, CSF1 may dampen the microglial response to LPS [10]. Withdrawal of CSF1 in cultured microglia up-regulated the pro-inflammatory cytokine IL-12 in response to LPS [25]. The *CSF1* rs1058885T allele resulted in an amino acid substitution from leucine to proline at position 408 (p. L408P). This protein variant demonstrated that lower CSF1 activity is required to stimulate formation of macrophage colonies in vitro compared to C-terminally truncated CSF1 [26]. Therefore, we proposed that the rs1058885 T allele may demonstrate protective effects against PD via down-regulation of CSF1 activity. Further functional studies to test the effect of this allele on microglia are necessary to explore the mechanism and the potential therapeutic strategies regarding targeting CSF1 in PD.

Mutations of *CSF1R* cause inherited diffuse white matter encephalopathy with spheroids pathology, an autosomal dominant neurodegenerative disorder presenting with such clinical features as parkinsonism, cognitive decline, and personality and behavioral changes [27]. Remarkably, mutations in CSF1R (p.P54Q, p.L536V, p.L868R, p.Q691H, and p.H703Y) have been reported in AD patients [28,29]. Our results failed to demonstrate a significant association between *CSF1R* rs10079250 and PD. However, the minor allele frequencies of rs10079250 demonstrated in our results (32.2%) and the East Asian population (38%) are higher than those observed in other populations (7%–11%) (http://www.1000genomes.org/home). This genetic discrepancy among different ethnicities may underestimate its significance regarding its association with disease. Given the high genetic overlap between different neurodegenerative diseases, further studies to assess the association between more genetic variants of *CSF1R* and PD or other neurodegenerative diseases are warranted.

Our study provides important information regarding the association of *CSF1* rs1058885 with PD patients in Taiwan. Study limitations include the relatively small sample size for the EOPD patients, which may have reduced the statistical power. Since the T allele in rs1058885 may be associated with a reduced risk of periodontitis [14], co-morbidity with periodontitis may affect the rs1058885 distribution in each group. However, the proportion of periodontitis in our cohort was not available, so our statistical results could not be adjusted according to periodontitis incidence. The potential interactions of environmental factors, such as exposures to smoking, toxins, heavy metals, or pesticides, with tested genetic variants were not explored. Future prospective studies involving different potential confounding factors in populations of different ethnicities are merited to confirm the potential association between CSF1/CSF1R signaling and PD or other neurodegenerative diseases.

## Figures and Tables

**Table 1:** Demographics and clinical characteristics of Parkinson’s disease (PD) patients and control subjects.

	PD	Controls	Total	*p* Value
Number	502	511	1013	
Age (years)	63.64 ± 10.76 (age at onset)	63.38 ± 11.90	63.51 ± 11.35	0.72
Gender (female/male)	253/249	252/259	505/508	0.73
Hohn and Yahr stage				
I	170 (33.9%)			
II	197 (39.2%)			
III	101 (20.1%)			
IV	25 (5.0%)			
V	9 (1.8%)			

**Table 2:** Genotype and allele frequencies of *CSF1* rs1058885 polymorphism among Parkinson’s disease (PD) patients and control subjects in Taiwan.

	PD (%)	Controls (%)	OR (95% CI)	*p* Value
Overall	502	511		
Genotype frequency				
CC	184 (36.7%)	155 (30.3%)	1.00	
CT	247 (49.2%)	261 (51.1%)	0.80 (0.61–1.05)	0.107
TT	71 (14.1%)	95 (18.6%)	0.63 (0.43–0.92)	0.015
Dominant model				
CC	184 (36.7%)	155 (30.3%)	1.00	
CT + TT	318 (63.3%)	356 (69.7%)	0.75 (0.58–0.98)	0.033
Recessive model				
CT + CC	431 (85.9%)	416 (81.4%)	1.00	
TT	71 (14.1%)	95 (18.6%)	0.72 (0.52–1.01)	0.056
Allele frequency				
Major allele (C)	615 (61.3%)	571 (55.9%)	1.00	
Minor allele (T)	389 (38.7%)	451 (44.1%)	0.80 (0.67–0.96)	0.014
**EOPD**	60	78		
Genotype frequency				
CC	27 (45.0%)	24 (30.8%)	1.00	
CT	27 (45.0%)	43 (55.1%)	0.56 (0.27–1.16)	0.116
TT	6 (10.0%)	11 (14.1%)	0.48 (0.16–1.51)	0.208
Allele frequency				
Major allele (C)	81 (67.5%)	91 (58.3%)	1.00	
Minor allele (T)	39 (32.5%)	65 (41.7%)	0.67 (0.41–1.11)	0.119
**LOPD**	442	433		
Genotype frequency				
CC	157 (35.5%)	131 (30.3%)	1.00	
CT	220 (49.8%)	218 (50.3%)	0.84 (0.63–1.13)	0.259
TT	65 (14.7%)	84 (19.4%)	0.65 (0.43–0.96)	0.031
Allele frequency				
Major allele (C)	534 (60.4%)	480 (55.4%)	1.00	
Minor allele (T)	350 (39.6%)	386 (44.6%)	0.82 (0.67–0.99)	0.035
**Female**	253	252		
Genotype frequency				
CC	89 (35.2%)	75 (29.8%)	1.00	
CT	128 (50.6%)	130 (51.6%)	0.83 (0.56–1.23)	0.351
TT	36 (14.2%)	47 (18.7%)	0.65 (0.38–1.10)	0.106
Allele frequency				
Major allele (C)	306 (60.5%)	280 (55.6%)	1.00	
Minor allele (T)	200 (39.5%)	224 (44.4%)	0.82 (0.64–1.05)	0.113
**Male**	249	259		
Genotype frequency				
CC	95 (38.2%)	80 (30.9%)	1.00	
CT	119 (47.8%)	131 (50.6%)	0.77 (0.52–1.13)	0.175
TT	35 (14.1%)	48 (18.5%)	0.61 (0.36–1.04)	0.069
Allele frequency				
Major allele (C)	309 (62.0%)	291 (56.2%)	1.00	
Minor allele (T)	189 (38.0%)	227 (43.8%)	0.78 (0.61–1.01)	0.057

CI: Confidence interval; EOPD: early-onset Parkinson’s disease; LOPD: late-onset Parkinson’s disease; OR: Odds ratio.

**Table 3:** Genotype and allele frequencies of the *CSF1R* rs10079250 polymorphism among Parkinson’s disease (PD) patients and control subjects in Taiwan.

	PD (%)	Controls (%)	OR (95% CI)	*p* Value
Overall	502	511		
Genotype frequency				
TT	199 (39.6%)	188 (36.8%)	1.00	
CT	233 (46.4%)	241 (47.2%)	0.91 (0.70–1.20)	0.509
CC	70 (13.9%)	82 (15.9%)	0.81 (0.55–1.18)	0.263
Dominant model				
TT	199 (39.6%)	188 (36.8%)	1.00	
CT + CC	303 (60.4%)	323 (63.2%)	0.89 (0.69–1.14)	0.351
Recessive model				
CT + TT	432 (86.1%)	429 (84.0%)	1.00	
CC	70 (13.9%)	82 (16.0%)	0.85 (0.60–1.20)	0.349
Allele frequency				
Major allele (T)	631 (62.8%)	617 (67.8%)	1.00	
Minor allele (C)	373 (37.2%)	405 (39.6%)	0.90 (0.75–1.08)	0.253
**EOPD**	60	78		
Genotype frequency				
TT	18 (30.0%)	27 (34.6%)	1.00	
CT	33 (55.0%)	33 (42.3%)	1.50 (0.70–3.23)	0.301
CC	9 (15.0%)	18 (23.1%)	0.75 (0.28–2.03)	0.572
Allele frequency				
Major allele (T)	69 (57.5%)	87 (55.8%)	1.00	
Minor allele (C)	51 (42.5%)	69 (44.2%)	0.93 (0.58–1.51)	0.774
**LOPD**	442	433		
Genotype frequency				
TT	181 (41.0%)	161 (37.2%)	1.00	
CT	200 (45.2%)	207 (47.8%)	0.86 (0.64–1.15)	0.304
CC	61 (13.8%)	65 (15.0%)	0.83 (0.55–1.26)	0.386
Allele frequency				
Major allele (T)	562 (63.6%)	529 (61.1%)	1.00	
Minor allele (C)	322 (36.4%)	337 (38.9%)	0.90 (0.74–1.09)	0.284
**Female**	253	252		
Genotype frequency				
TT	94 (37.2%)	92 (36.5%)	1.00	
CT	122 (48.2%)	120 (47.6%)	1.00 (0.68–1.46)	0.980
CC	37 (14.6%)	40 (15.9%)	0.91 (0.53–1.54)	0.713
Allele frequency				
Major allele (T)	310 (61.3%)	304 (60.3%)	1.00	
Minor allele (C)	196 (38.7%)	200 (39.7%)	0.96 (0.75–1.24)	0.758
**Male**	249	259		
Genotype frequency				
TT	105 (42.2%)	96 (37.1%)	1.00	
CT	111 (44.6%)	121 (46.7%)	0.84 (0.57–1.22)	0.362
CC	33 (13.3%)	42 (16.2%)	0.72 (0.42–1.23)	0.224
Allele frequency				
Major allele (T)	321 (64.5%)	313 (60.4%)	1.00	
Minor allele (C)	177 (35.5%)	205 (39.6%)	0.84 (0.65–1.09)	0.185

CI: Confidence interval; EOPD: early-onset Parkinson’s disease; LOPD: late-onset Parkinson’s disease; OR: Odds ratio.
